# Beta‐lactam allergies, surgical site infections, and prophylaxis in solid organ transplant recipients at a single center: A retrospective cohort study

**DOI:** 10.1111/tid.13907

**Published:** 2022-10-18

**Authors:** Clayton Mowrer, Elizabeth Lyden, Stephen Matthews, Anum Abbas, Scott Bergman, Bryan T. Alexander, Trevor C. Van Schooneveld, Erica J. Stohs

**Affiliations:** ^1^ Division of Infectious Diseases Department of Medicine University of Nebraska Medical Center Omaha Nebraska USA; ^2^ Department of Biostatistics College of Public Health University of Nebraska Medical Center Omaha Nebraska USA; ^3^ Infection Control and Epidemiology Nebraska Medicine Omaha Nebraska USA; ^4^ Department of Pharmaceutical and Nutritional Care Nebraska Medicine Omaha Nebraska USA

**Keywords:** antibiotic allergy, surgical site infections, surgical site infection prophylaxis, solid organ transplant

## Abstract

**Background:**

Beta‐lactam allergies (BLAs) are common in hospitalized patients, including transplant recipients. BLA is associated with decreased use of preferred surgical site infection (SSI) prophylaxis and increased SSIs, but this has not been studied in the transplant population.

**Methods:**

We reviewed adult heart, kidney, and liver transplant recipients between January 1, 2016 and December 31, 2019 to characterize reported BLA and collect SSI prophylaxis regimens at time of transplant. We compared the use of preferred SSI prophylaxis and SSI incidence based on reported BLA status. Post hoc we collected antibiotic days of therapy (DOT) (excluding pneumocystis prophylaxis) in the 30‐day period posttransplant for patients without SSI. We utilized descriptive statistics for comparisons.

**Results:**

Of 691 patients included (116 heart, 400 kidney, and 175 liver transplant recipients), 118 (17%) reported BLA. Rash and hives were the two most reported BLA reactions (36% and 24%), categorized as potential T‐cell mediated and IgE mediated, respectively. Preferred SSI prophylaxis was prescribed in 13 (11%) patients with BLA and 573 (92%) without BLA (*p* < .001). No difference could be detected in SSI incidence between BLA and non‐BLA patients (4.2 vs. 4.3%, *p* = 1.0). Of 659 without SSI, 169 (25.6%) received antibiotics within 30 days of transplant; mean antibiotic DOT for BLA and non‐BLA patients were 3.5 ± 8.0 versus 2.3 ± 5.8, *p* = .12.

**Conclusion:**

BLA transplant recipients received nonpreferred SSI prophylaxis more frequently than non‐BLA recipients, but there was no difference in 30‐day SSIs between the groups. One‐fourth of solid organ transplant recipients received systemic antibiotics within 30 days of transplant.

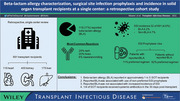

**Abbreviations**
BLA: beta‐lactam allergy
CDC NHSN: Centers for Disease Control and Prevention National Healthcare Safety Network
SOT: solid organ transplant
SSI: surgical site infections

## 1 INTRODUCTION

Beta‐lactam allergies (BLA) are reported in approximately 20% of all hospitalized patients.^1–7^ Inaccurate antibiotic allergy labels have been associated with increased use of inappropriate antibiotics, such as more broad‐spectrum regimens with a higher likelihood for toxicity and adverse clinical outcomes, including increased rate of *Clostridioides difficile* infection, surgical site infections (SSI), and infections with resistant organisms.^1,3,8–11^ Limited data are available for solid organ transplant (SOT) recipients with BLA labels; one center reported a prevalence of 16% at time of transplant with increased use of nonbeta‐lactam antibiotics during their transplant course.^1^

SSIs cause significant morbidity and mortality, along with substantial financial burden.^12^, ^13^ In order to reduce the incidence of SSIs, current guidelines recommend the use of beta‐lactams for preoperative SSI prophylaxis.^14^ Use of alternative nonbeta‐lactam surgical prophylaxis has been associated with increased risk of SSI,^9^, ^15^, ^16^ although no studies have been performed in SOT recipients. No studies have examined SSI prophylaxis utilization in SOT recipients with BLA. We sought to assess our institution's prevalence of reported BLA at transplantation while characterizing these allergies and then assess use of SSI antibiotic prophylaxis and incidence of posttransplant SSIs based on BLA status.

## 2 METHODS

### 2.1 Study design

We retrospectively reviewed patients undergoing heart, kidney, and liver transplantation at the Nebraska Medical Center between January 1, 2017 and December 31, 2019. We characterized BLA status at the time of transplant and performed a cohort analysis to evaluate the association of BLA status with use of preferred SSI antibiotic prophylaxis during transplantation and 30‐day SSI incidence.

### 2.2 Study Subjects

First‐time recipients of heart, kidney, and liver transplantations aged 18 years or older at the time of transplantation were included. Exclusion criteria included recipients of combined transplantations. Transplant recipients receiving antibiotics for a preceding or known donor‐derived infection at the time of transplant were excluded from analysis of preferred SSI prophylaxis and SSI incidence as pretransplant antibiotic could alter SSI prophylaxis selections.

### 2.3 Definitions

BLA status was defined as reported allergies to penicillins, cephalosporins, and/or carbapenems documented in the electronic medical record on the day of transplantation. Reported reactions were categorized as immediate (potential IgE‐mediated) reactions, delayed (potential T‐cell‐mediated), nonallergic reactions, and not otherwise specified based on previously described criteria.^6^, ^17^, 

Primary outcomes were 30‐day SSI, as defined per Centers for Disease Control and Prevention National Healthcare Safety Network (NHSN) criteria,^18^ and receipt of preferred SSI prophylaxis, defined as first‐line antimicrobials per institution and organ‐based SSI prevention protocols. Preferred agents for heart transplant recipients included cefazolin 2 g IV every 8 h and vancomycin 15 mg/kg IV every 12 h preoperatively through 48 h posttransplant, cefazolin 1 g IV every 12 h preoperatively through 24 h posttransplant for kidney transplant recipients, and ampicillin‐sulbactam 3 g IV every 8 h preoperatively through 24 h posttransplant for liver transplant recipients. Our local antimicrobial stewardship program developed alternative SSI prophylaxis regimens for penicillin‐allergic patients, which included levofloxacin and vancomycin for heart transplant recipients, aztreonam, and clindamycin for kidney transplant recipients, and aztreonam and vancomycin for liver transplant recipients. Notably, shortages of preferred SSI prophylactic agents occurred during the study period: cefazolin between October 2018 and December 2019 and ampicillin‐sulbactam from June through the remainder of 2019. Piperacillin‐tazobactam was recommended in place of ampicillin‐sulbactam; cefazolin use was prioritized for the use SSI prophylaxis and continued to be used in this population despite shortages.

In order to evaluate if nonprophylactic antibiotic use posttransplant contributed to SSI incidence, 30‐day posttransplant antibiotic utilization in patients who did not develop an SSI was pursued as a post hoc secondary outcome. DOT were collected for the following agents: cefazolin, cephalexin, ceftriaxone, cefepime, fluoroquinolones, carbapenems, piperacillin‐tazobactam, ampicillin‐sulbactam, amoxicillin‐clavulanate, intravenous vancomycin, linezolid, doxycycline, daptomycin, clindamycin, aztreonam, and metronidazole. We excluded pneumocystis prophylaxis but could include SSI prophylaxis received for surgeries after transplantation in the 30‐day period. For renally adjusted antibiotics that may not be administered daily (i.e., vancomycin), start and stop dates were utilized to determine days of active therapy. Oral vancomycin was not captured due to lack of systemic absorption that would affect development of SSI. Inpatient and outpatient antibiotic DOT was abstracted from the electronic health record.

### 2.4 Data collection

SSI data including baseline patient characteristics required for submission to the CDC NHSN were collected by a certified infection preventionist, which were available in the institution's Infection Control and Prevention SSI database. All other data were abstracted from electronic medical records by manual review. The University of Nebraska Medical Center (UNMC) Institutional Review Board designated this work as quality improvement, exempt from institutional review board (IRB) review.

### 2.5 Data analysis

We utilized descriptive statistics including counts and percentages for categorical variables and means, standard deviations, medians for continuous data. Fisher's exact test was used to look at associations of categorical data between cohorts. The independent sample *t*‐test was used to compare continuous data between cohorts. Interquartile ranges were reported for median values. All analyses were performed using SAS, Version 9.4. *p* < .05 was considered statistically significant.

## 3 RESULTS

**FIGURE 1:**
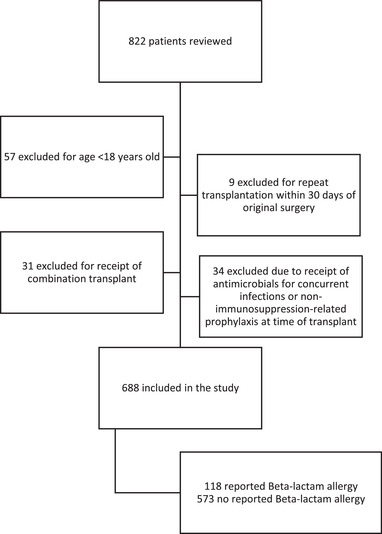
Patient flow diagram

A total of 822 patients were reviewed with 691 unique patients included after exclusion criteria were applied (Figure 1). Among the 691 SOT recipients evaluated, 118 (17%) reported a BLA. Table 1 reports baseline patient characteristics from CDC NHSN by BLA status. Significantly more females than males reported a BLA (67 [57%] versus 206 [36%], *p* < .001). There was no difference in mean age, diabetes mellitus, body mass index, type of solid organ transplant, or year transplanted. Kidney transplant recipients made up over half (*n* = 400, 58%) of the entire cohort, while liver and heart transplant recipients represented the remainder (*n* = 175, 25% and *n* = 116, 17%, respectively).

**TABLE 1:** Baseline CDC NHSN patient characteristics by reported BLA

Patient characteristics	Reported BLA (*n* = 118)	No reported BLA (*n* = 573)	*p*‐Value
Age in years, mean ± SD	54.7 ± 12.0	54.6 ± 12.7	.93
Age in years, median (IQR)	57.0 (48.0–64.0)	58.0 (47.0–64.0)	
Female, *n* (%)	67 (56.8)	206 (36.0)	<.0001
Comorbidities			
Diabetes mellitus, *n* (%)	47 (39.8)	228 (39.8)	1.0
BMI, median (IQR)	28.5 (24.8–32.9)	28.3 (25.1–32.6)	.72
SOT, *n* (%)			.36
Heart	24 (20.3)	92 (16.1)	
Kidney	62 (52.5)	338 (59.0)	
Liver	32 (27.1)	143 (25.0)	
Year transplanted, *n* (%)			.66
2017	38 (32.2)	205 (35.8)	
2018	34 (28.8)	169 (29.5)	
2019	46 (39.0)	199 (34.7)	

Abbreviations: CDC, Centers for Disease Control and Prevention; NHSN, National Healthcare Safety Network; BLA, β‐lactam allergy; BMI, body mass index; SD, standard deviation; SOT, solid organ transplant.

Antibiotic allergy reactions among the 118 patients reporting BLAs are characterized in Table 2. They reported a total of 125 BLAs, 101 (81%) being penicillins, 24 (19%) cephalosporins, and 1 (0.8%) carbapenem. Rash was the most reported BLA reaction (45/125 [36%]), followed by hives (30/125 [24%]). Fifty‐two of the reactions were classified as immediate/potential IgE mediated and 52 as delayed/potential T‐cell mediated. Nonallergic and unspecified reactions comprised 28% (35/125) and 9% (11/125) of the reactions, respectively.

**TABLE 2:** Reported antibiotic reactions by class among 118 β‐lactam allergy (BLA) transplant recipients^†^

Reported reactions	Penicillins	Cephalosporins	Carbapenems
*N*	101	24	1
Immediate (potential IgE mediated), total	44	8	0
Hives	25	5	0
Anaphylaxis	6	0	0
Angioedema	6	1	0
Shortness of breath	3	1	0
Swelling	2	1	0
Hypotension	1	0	0
Apnea	1	0	0
Delayed (potential T‐cell mediated), total	42	9	1
Rash	36	8	1
Itching	6	1	0
Nonallergic, total	25	9	1
Nausea/vomiting	8	3	0
Diarrhea	3	2	0
Palpitations	2	0	0
Yeast infection	2	0	0
Oral blisters	2	0	0
Abdominal pain	1	1	0
Tight chest	1	0	1
Fever	1	1	0
Other, total	5	2	0
Not specified, total	8	3	0

† Eight patients reported allergies to more than 1 class of beta‐lactam antibiotics. Patients may have listed more than one reaction for a reported BLA. “Other” reactions included singular reports of disease of the liver, shock, mood disturbance, muscle pain, and seizure in the penicillin group; and rectal bleeding, hypertension in the cephalosporin group.

Preferred SSI prophylaxis was prescribed in 13 of 118 (11%) BLA patients and 529 of 573 (92.3%) non‐BLA patients (*p* < .0001). Table 3 breaks down SSI antibiotics administered by organ transplant. Vancomycin plus levofloxacin was most commonly given to BLA heart transplant recipients, aztreonam plus clindamycin to BLA kidney transplant recipients, and vancomycin plus aztreonam to BLA liver transplant recipients. 19 patients (6 heart, 5 kidney, and 8 liver transplant recipients) with reported BLA received a beta‐lactam as part of SSI prophylaxis. All but four liver recipients were given a beta‐lactam from a different class than their reported allergy. Notably, four of the liver transplant recipients with reported penicillin allergy received piperacillin‐tazobactam or ampicillin‐sulbactam. One of these patients underwent de‐sensitization guided by the Transplant Infectious Diseases service; the rest tolerated intra‐operative administration of a penicillin derivative without reported adverse events. Non‐BLA transplant recipients largely received institutional preferred SSI regimens. Deviances from preferred SSI regimens received by non‐BLA patients included ceftriaxone in 22% of kidney transplant recipients and piperacillin‐tazobactam in 32% of liver transplant recipients.

**TABLE 3:** Surgical site infection (SSI) prophylaxis use by β‐lactam allergy (BLA) status and organ transplant

SSI prophylaxis	Reported BLA *n* (%)	No reported BLA *n* (%)	*p*‐Value
Preferred agents	13/118 (11.0)	529/573 (92.3)	<.0001

Heart transplant	(*n* = 24)	(*n* = 92)	
Cefazolin^†^	6 (25)	85 (92.4)	<.0001
Vancomycin^†^	24 (100)	91 (98.9)	1.0000
Levofloxacin^‡^	17 (70.8)	3 (3.3)	<.0001
Cefuroxime	0 (0.0)	7 (7.6)	.3419
Ceftriaxone	0 (0.0)	1 (1.1)	1.0000
Pip‐tazo	1 (4.2)	0 (0.0)	.2069
Clindamycin	1 (4.2)	0 (0.0)	.2069
Cefepime	0 (0.0)	1 (1.1)	1.0000

Kidney transplant	(n = 62)	(n = 338)	
Cefazolin^†^	5 (8.1)	331 (97.9)	<.0001
Aztreonam^‡^	59 (95.2)	4 (1.2)	<.0001
Clindamycin^‡^	59 (95.2)	4 (1.2)	<.0001
Ceftriaxone	1 (1.6)	76 (22.5)	<.0001
Cefuroxime	0 (0)	13 (3.9)	.2340
Cefoxitin	0 (0)	8 (2.4)	0.6155
Ampicillin‐sulbactam	0 (0)	4 (1.2)	1.0000
Piperacillin‐tazobactam	0 (0)	3 (0.9)	1.0000
Vancomycin	2 (3.2)	2 (0.6)	.1152
Oxacillin	0 (0)	1 (0.3)	1.0000

Liver transplant	(*n* = 32)	(*n* = 143)	
Ampicillin‐sulbactam^†^	2 (6.3)	114 (79.7)	<.0001
Piperacillin‐tazobactam	5 (15.6)	46 (32.2)	.0843
Aztreonam^‡^	27 (84.4)	5 (3.5)	<.0001
Vancomycin^‡^	26 (81.3)	4 (2.8)	<.0001
Cefoxitin	1 (3.1)	5 (3.5)	1.0000
Cefazolin	0 (0.0)	4 (2.8)	1.0000
Ceftriaxone	0 (0.0)	1 (0.7)	1.0000
Metronidazole	0 (0.0)	1 (0.7)	1.0000

† Preferred SSI prophylaxis agents.

‡ Recommended SSI prophylaxis in case of severe penicillin allergy regimen.

SSIs occurred in 32 of 691 transplant recipients (incidence of 4.6%) within 30 days of transplantation. Intraabdominal infections represented 70% of SSIs, while primary superficial infections made up 20%. SSIs occurred in 7/116 (6%), 7/400 (2%), and 18/175 (10%) of heart, kidney, and liver transplant recipients, respectively. Causative organisms were identified in half of the SSIs, some of which were polymicrobial. Vancomycin‐resistant enterococci was the most identified organism (17% of SSIs) and was found exclusively in liver transplant recipients. There was no difference in SSI incidence between the BLA versus non‐BLA groups (4.2 vs 4.7%, *p* = 1.0). Table 4 demonstrates breakdown of SSI characterization between BLA versus non‐BLA patients. Because the exclusion of patients receiving antibiotics prior to transplant may affect incidence of SSIs, post hoc sensitivity analysis of SSIs when 33 of these patients were included still found no difference in SSI incidence between BLA and non‐BLA patients (5/124 [4.0%] versus 27/601 [4.5%], *p* = 1.0).

**TABLE 4:** Surgical site infection (SSI) characterization of 32 SSIs based on patient‐reported β‐lactam allergy (BLA) status

SSI	BLA	No BLA	*p*‐Value
*N* (%)	5 (4.2)	27 (4.7)	1.0000
By Transplanted Organ			NS
Heart	2 (8.3)	5 (5.4)	
Kidney	0 (0.0)	7 (2.1)	
Liver	3 (9.4)	15 (10.5)	
SSI classification			.7468
Intra‐abdominal	3 (2.5)	18 (3.1)	
Superficial infection, primary	1 (0.9)	5 (0.9)	
Mediastinitis	0 (0.0)	2 (0.4)	
Lower respiratory infection	1 (0.9)	1 (0.2)	
Deep infection, primary	0 (0.0)	1 (0.2)	
Organism identified	3 (60.0)	12 (44.4)	.6454
*Pseudomonas aeruginosa*	0 (0.0)	2 (7.4)	
*Serratia marscecens*	0 (0.0)	1 (3.7)	
*E. coli* ^†^	0 (0.0)	1 (3.7)	
*Klebsiella pneumoniae* ^†^	0 (0.0)	1 (3.7)	
*Enterobacter cloacae*	0 (0.0)	1 (3.7)	
*Enterobacter aerogenes*	0 (0.0)	1 (3.7)	
*Staphylococcus aureus*	2 (40.0)	0 (0.0)	
*Enterococcus faecalis*	0 (0.0)	3 (11.1)	
*Enterococcus faecium* ^‡^	1 (20.0)	4 (14.8)	
Viridans group *Streptococcus*	0 (0.0)	1 (3.7)	
*Candida albicans/dubliensis*	1 (20.0)	1 (3.7)	
*Candida glabrata*	0 (0.0)	1 (3.7)	
*Candida krusei*	0 (0.0)	1 (3.7)	
No organism identified	2 (40.0)	15 (55.6)	

Abbreviations: BLA, β‐lactam allergy; SSI, surgical site infection; NS, not significant; SD, standard deviation; UNMC, University of Nebraska Medical Center; IRB, institutional review board.

†One isolate of *E. coli* and *Klebsiella pneumoniae* were identified as having extended spectrum beta‐lactamase.

‡Each *E. faecium* isolate was vancomycin resistant.

Post hoc antibiotic DOT in the 30‐day posttransplant period in the 659 patients who did not develop SSI is displayed in Table 5. Excluding SSI and pneumocystis prophylaxis, 169 (25.6%) received at least one antibiotic day of therapy within 30 days posttransplant, and there was no difference between patients reporting BLA versus not (31/113 vs. 138/546 [27.4% vs. 25.3%], *p* = .64). Mean total antibiotic DOT was higher in patients reporting BLA (3.5 ± 8.0 vs. 2.3 ± 5.8), but this was not statistically significant (*p* = .12). Liver transplant recipients had the highest mean antibiotic DOT (5.6 ± 11.4 in BLA vs. 4.1 ± 8.0 in non‐BLA) among the organ groups. Mean aztreonam DOT was higher in BLA compared to non‐BLA patients (0.12 ± 0.7 vs. 0.0 ± 0), although this did not meet threshold for significance (*p* = .056). There was a trend toward higher mean daptomycin DOT in the BLA versus non BLA group (0.43 ± 2.3 vs. 0.05 ± 0.9, *p* = .088). Mean DOT for other antibiotics was not significant or equivalent for all other antibiotics except for cephalexin. Mean cephalexin DOT was statistically significantly higher in the non‐BLA versus BLA group (0.11 ± 0.8 vs. 0 ± 0, *p* < .01).

**TABLE 5:** Antibiotic days of therapy in 30‐day post‐transplant by patients who did not have an Surgical site infection (SSI) by β‐lactam allergy (BLA) status

Antibiotic use	Reported BLA (*n* = 113)	No reported BLA (*n* = 546)	*p*‐Value
Any posttransplant antibiotic use, *n* (%)	31 (27.4)	138 (25.3)	.6370
Days of antibiotics used, mean ± SD (range)	3.5 ± 8.0 (0–45)	2.3 ± 5.8 (0–50)	.12
Heart transplant	2.4 ± 4.9 (0–15)	3.5 ± 7.4 (0–50)	
Kidney transplant	3.0 ± 6.9 (0–31)	1.3 ± 3.8 (0–36)	
Liver transplant	5.6 ± 11.4 (0–45)	4.1 ± 8.0 (0–43)	
Vancomycin	0.45 ± 1.92 (0–17)	0.32 ± 1.7 (0–20)	.49
Cefazolin	0.12 ± 0.8 (0–7)	0.07 ± 0.5 (0–9)	.60
Ceftriaxone	0.07 ± 0.7 (0–7)	0.22 ± 1.5 (0–22)	.093
Cefepime	0.34 ± 1.4 (0–8)	0.18 ± 1.0 (0–10)	.27
Fluoroquinolone	0.38 ± 2.7 (0–27)	0.39 ± 1.9 (0–18)	.99
Carbapenem	0.26 ± 1.6 (0–15)	0.14 ± 1.1 (0–16)	.46
Piperacillin‐tazobactam	0.52 ± 1.9 (0–10)	0.52 ± 1.8 (0–16)	.97
Ampicillin‐sulbactam	0.11 ± 1.0 (0–11)	0.07 ± 0.7 (0–12)	.75
Amoxicillin‐clavulanate	0.30 ± 2.4 (0–24)	0.04 ± 0.4 (0–6)	.26
Doxycycline	0.19 ± 1.3 (0–11)	0.09 ± 1.0 (0–12)	.49
Linezolid	0.13 ± 0.9 (0–8)	0.05 ± 0.6 (0–9)	.36
Metronidazole	0.12 ± 0.5 (0–3)	0.05 ± 0.4 (0–7)	.19
Aztreonam	0.12 ± 0.7 (0–6)	0.0 ± 0 (0–0)	.056
Cephalexin	0 ± 0 (0–0)	0.11 ± 0.8 (0–8)	.0013*
Clindamycin	0.01 ± 0.1 (0–1)	0.01 ± 0.2 (0–4)	.89
Daptomycin	0.43 ± 2.3 (0–17)	0.05 ± 0.9 (0–17)	.088

## 4 DISCUSSION

To our knowledge, this is the first study to assess the impact of BLA status on SSI prophylaxis use and incidence of SSIs in the SOT population. In line with prior studies, BLA status was associated with the use of nonpreferred SSI prophylaxis in our SOT population^9^, ^15^; however, it was not associated with increased incidence of SSIs in our study. Low overall SSI incidence likely precluded detection of a statistical difference between cohorts. There may be several reasons for this. A major contributor is the finding that nearly one quarter of all transplant recipients received nonprophylactic systemic antibiotics within 30 days of transplantation. Another factor may be the use of broadened SSI prophylaxis from the preferred regimen during the prophylaxis period. For example, in kidney transplant recipients, who represented over half of our study population, approximately one‐fifth received broadened prophylaxis with ceftriaxone rather than the recommended cefazolin. Decisions to deviate from standard SSI prophylaxis were often not well documented and thus difficult to ascertain. We surmise that alterations in perioperative prophylaxis may be directed toward known donor or recipient urinary colonization prior to transplant.^19^, ^20^, ^21^ Interestingly the SSI incidence among kidney transplant recipients in our cohort (2%) was lower than nationally reported ranges of 3%–11%; meanwhile, SSI incidence in heart and liver transplant recipients was comparable to nationally reported incidences of 4%–19% and 10%–37%, respectively.^21^, ^22^, ^23^, ^24^ Finally, in our study population, providers were willing to prescribe beta‐lactams in patients who reported BLA.

Reported penicillin allergy predominated the BLA cohort (>85%), but this did not seem to preclude cephalosporin and carbapenem use in BLA patients in their posttransplant period, which may reflect growing provider acceptance of low cross‐reactivity between BLA classes.^25^ This was supported by review of our data in which one of 8 patients with reported BLA received beta‐lactams as part of their SSI prophylaxis, most from a different BLA class. The three liver transplant recipients with reported penicillin allergy that were challenged intra‐operatively with a penicillin‐derivative did well without any reported reactions. Notably, these patients received high‐dose methylprednisone as part of induction immunosuppresion, which may have blunted a systemic response.

Data have demonstrated that the use of cefazolin is safe and appropriate in most patients with a penicillin allergy.^26^, ^27^, ^28^, ^29^ With this in mind, our antimicrobial stewardship program has encouraged the use of cefazolin as SSI prophylaxis for penicillin‐allergic patients since July 2017. In addition, our antimicrobial stewardship team routinely reviews SSI prophylaxis regimens, which includes noncefazolin‐based regimens, to assure that alternative regimens provide comparable antimicrobial coverage. This may also contribute to low SSI rates in our study population.

Opportunities to optimize BLA characterization and de‐label incorrect antibiotic allergies exist in our transplant population. Overall, reported BLAs were present in 17% of our solid organ transplant population, which is similar to the prevalence in both hospitalized persons in the general population and the transplant population at another medical center.^1^, ^2^, ^3^ However, data have demonstrated that true drug allergies are much less prevalent than reported by patients. In fact, the American Academy of Allergy, Asthma, and Immunology have shown that true penicillin allergies occur in <1% of the general population, and that even in those with proven allergy, sensitivity to the agent wanes over time.^6^ Furthermore, in studies implementing antibiotic allergy testing, up to 83% of patients with reported allergies did not exhibit positive testing and could be successfully delabeled.^4^ In our BLA cohort, we utilized a previously described categorization method based on reported reaction to help determine the likelihood of an antibiotic allergy.^6^, ^17^ Rash and hives were the most commonly reported reactions, categorized as potential T‐cell mediated and IgE mediated reactions, respectively. This is similar to what has been reported in the general population.^17^ In our study, determining the likelihood of true immune response was limited by chart review, where time since reported reaction and further characterization of said *rash* was not possible without direct patient inquiry. In addition, one quarter of patients with nonallergic BLA reactions represent potential opportunities for allergy delabeling in our SOT population.^17^

There were no statistically significant differences in antibiotic DOT in the 30‐day posttransplant period between BLA and non‐BLA SOT recipients in our study, although BLA patients demonstrated trends of increased DOT of aztreonam and daptomycin. Our study was not designed or powered to compare antibiotic DOT. However, in a study of over 2000 inpatient SOT and hematopoietic cell transplant recipients, BLA SOT recipients had significantly higher DOT for vancomycin, clindamycin, aztreonam, and fluoroquinolones in the first 100 days posttransplant.^1^ Extending our posttransplant period for DOT collection to 100 days or more may allow for appropriate comparisons.

Optimized BLA status in transplant recipients remains a worthy antimicrobial stewardship goal as beta‐lactams are less toxic and costly than alternative antibiotics.^10^, ^30^ While our study did not find an association between reported BLA status and SSI incidence, it did demonstrate substantial antimicrobial utilization in transplant recipients in the first month following transplantion. SOT recipients experience a variety of complications postoperatively, and, as a result of chronic immunosuppression, this population is at a higher risk of infection and often receives more antimicrobials than the general population.^31^, ^32^ Inaccurate antibiotic labels are associated with administration of inappropriate antibiotic regimens with increased risk for toxicity and adverse clinical outcomes, which carry significant morbidity and mortality.^1^, ^3^, ^8^, ^9^, ^10^, ^11^, ^12^, ^13^ In addition, second‐line or broadened SSI prophylaxis may increase the risk of antimicrobial resistance posttransplant.^20^, ^31^ Therefore, in a population exposed to an increased number of antimicrobials, ensuring the receipt of the most appropriate regimen is crucial.

There are several limitations to this study. First, this was a retrospective, single‐center study, limiting generalization. Second, shortages of preferred SSI prophylactic agents occurred during the study. However, this would affect the non‐BLA group principally, and bias preferred SSI prophylaxis toward the null hypothesis. Third, strict exclusion criteria, including those receiving systemic antibiotics for preceding or donor‐derived infection, may have limited SSIs. However, in a post hoc analysis including those who were initially excluded for that reason did not reveal a difference in SSIs between BLA and non‐BLA cohorts. Fourth, implementation of guidelines for the use of alternative agents in patients with antibiotic allergies, along with the education of antibiotic allergies, may reflect use in reported BLA recipients, but this would bias preferred SSI use toward the null hypothesis. Finally, whether SSI incidence or 30‐day antibiotic use is associated with specific transplant parameters is not known. At present, such adjustments are not required by NHSN reporting.

## 5 CONCLUSION

We found that transplant recipients with reported BLA at the time of transplant received more nonpreferred SSI prophylactic antibiotic compared to non‐BLA transplant recipients, but they had similar 30‐day SSI incidences. Overall, one quarter of transplant recipients received systemic antibiotic therapy following the SSI prophylaxis within 30‐days of transplantation.

## CONFLICT OF INTEREST

Erica Stohs has received research support from Reviral, Ltd. Bryan Alexander is a member of Astellas Pharma Advisory Board. None of the associations mentioned are relevant to this article. Other authors report no conflict of interest relevant to this article.

## AUTHOR CONTRIBUTIONS

*Writing of the manuscript*: Clayton Mowrer and Erica J. Stohs. *Review of the manuscript*: Elizabeth Lyden, Trevor van Schooneveld, Scott Bergman, and Bryan Alexaner. *Study design and interpretation of the data*: Clayton Mowrer and Erica J. Stohs. *Data collection*: Clayton Mowrer, Stephen Matthews, Erica Stohs, and Anum Abbas. *Data analysis*: Clayton Mowrer, Erica Stohs, and Elizabeth Lyden.

## Supporting information



Graphical Abstract
