# Supplementary material for: Beta‐lactam allergies, surgical site infections, and prophylaxis in solid organ transplant recipients at a single center: A retrospective cohort study
Source: Transpl Infect Dis. 2022 Oct 18;24(5):e13907. doi: 10.1111/tid.13907 (PMC9787036; doi:10.1111/tid.13907)
Supplement: Supplementary file 1 — Graphical Abstract [file TID-24-e13907-s001.pptx]

## Slide 1
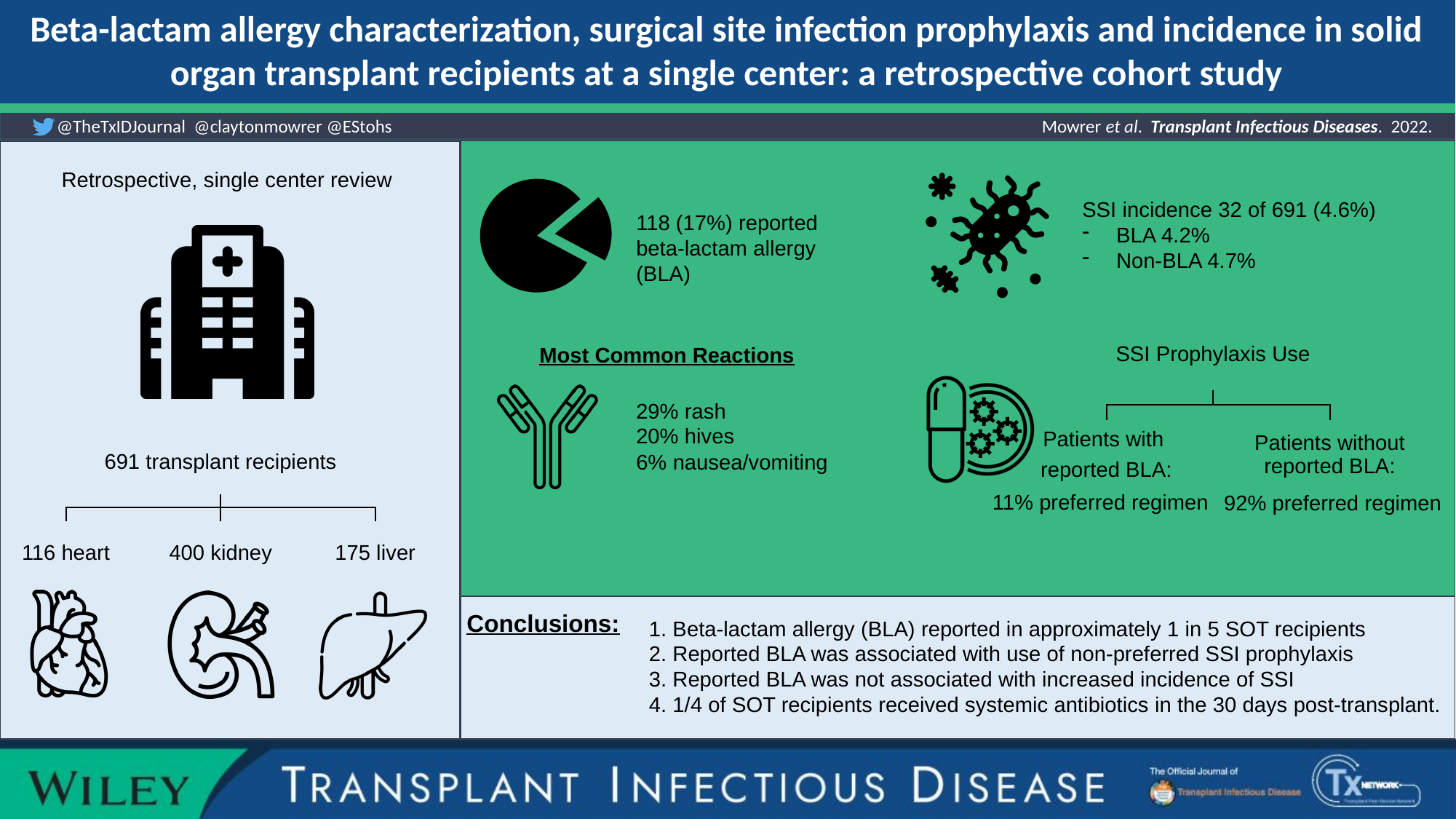

Beta-lactam allergy characterization, surgical site infection prophylaxis and incidence in solid organ transplant recipients at a single center: a retrospective cohort study
Mowrer et al. Transplant Infectious Diseases. 2022.
 @TheTxIDJournal @claytonmowrer @EStohs
### Chart
| Category | Sales |
|---|---|
| BLA | 0.17 |
| No BLA | 0.73 |
Retrospective, single center review
SSI incidence 32 of 691 (4.6%)
BLA 4.2%
Non-BLA 4.7%
118 (17%) reported beta-lactam allergy (BLA)
Most Common Reactions
29% rash
20% hives
6% nausea/vomiting
11% preferred regimen
92% preferred regimen
Conclusions:
1. Beta-lactam allergy (BLA) reported in approximately 1 in 5 SOT recipients
2. Reported BLA was associated with use of non-preferred SSI prophylaxis
3. Reported BLA was not associated with increased incidence of SSI
4. 1/4 of SOT recipients received systemic antibiotics in the 30 days post-transplant.
